# Acceptability Among Healthcare Providers of In Situ, Low-Dose, High-Frequency Neonatal Resuscitation Simulation Training Using Innovative Tools: Evidence from the Safer Births Bundle of Care

**DOI:** 10.3390/children12091150

**Published:** 2025-08-29

**Authors:** Florence Salvatory Kalabamu, Vickfarajaeli Daudi, Robert Deogratias Moshiro, Dunstan R. Bishanga, Benjamin Kamala, Paschal Mdoe, Hege Ersdal, Rose Mpembeni

**Affiliations:** 1 Department of Pediatrics and Child Health, Kairuki University, Dar es Salaam P.O. Box 65300, Tanzania; 2 School of Public Health and Social Sciences, Muhimbili University of Health and Allied Sciences, Dar es Salaam P.O. Box 65001, Tanzania; moshiror@gmail.com (R.D.M.); dbishanga@gmail.com (D.R.B.); kamala8086@gmail.com (B.K.); rcmpembeni@gmail.com (R.M.); 3 Haydom Lutheran Hospital, Haydom P.O. Box 9000, Tanzania; farajaeli@yahoo.com (V.D.); pfmdoe@gmail.com (P.M.); 4 Department of Pediatrics and Child Health, KCMC University, Moshi P.O. Box 2240, Tanzania; 5 Department of Pediatrics and Child Health, Muhimbili National Hospital, Dar es Salaam P.O. Box 65000, Tanzania; 6 Ifakara Health Institute, Dar es Salaam P.O. Box 70373, Tanzania; 7 Department of Anesthesia, Stavanger University Hospital, 4011 Stavanger, Norway; hege.ersdal@safer.net; 8 Faculty of Health Sciences, University of Stavanger, 4021 Stavanger, Norway

**Keywords:** acceptability, simulation, resuscitation, Neonatalie Live, NeoBeat

## Abstract

**Introduction**: Newborn mortality is unacceptably high, especially in low- and middle-income countries. The Safer Births Bundle of Care (SBBC) was implemented in Tanzania, including training of healthcare workers on neonatal resuscitation by means of frequent in situ simulation training using improved training tools. We aimed to assess the acceptability of this training model among healthcare providers in selected health facilities under SBBC intervention. **Methods**: A cross-sectional study was conducted among healthcare workers in labor wards and obstetric theaters in selected facilities one year after the introduction of the SBBC model. The theoretical framework for assessment of the acceptability of healthcare interventions was used to assess the acceptability of the training model and accompanying tools. The chi-square test was used to assess the association between acceptability in specific constructs and average individual practice per month, while a modified Poisson regression analysis was used to assess factors associated with acceptability in specific framework constructs. **Results**: A total of 227 healthcare workers were enrolled in the study. Overall, 223 (98.2%) accepted the intervention. However, 207 (91.2%) reported that the intervention increased their work burden, while 39 (17.2%) reported that it interfered with other equally important activities. The level of health facility was independently associated with the reporting that engaging in simulation practice interfered with other equally important activities. **Conclusions**: In situ, low-dose, high-frequency facility-based simulation training for neonatal resuscitation was highly acceptable among healthcare providers. However, the perceived increased work burden of this intervention and interference with other equally important activities were identified as potential threats to successful implementation.

## 1. Introduction

An estimated 2.3 million newborns die every year, with around 95% of these deaths occurring in low- and middle-income countries (LMIC) [1]. The main causes of death are the same globally, with the major culprits being complications of prematurity, birth asphyxia, and infections [2,3,4]. The risks for mortality are multifactorial; however, they are directly linked to suboptimal quality of care around the time of delivery [5].

Tanzania has recorded a remarkable improvement in reducing under-five and infant mortality; however, some reduction in newborn mortality was achieved, with the current estimation at 24 deaths per 1000 live births [6]. With the current annual reduction rate of newborn mortality, the possibility of attaining the Sustainable Development Goals (SDGs) target of reducing newborn mortality to 12 deaths or fewer per 1000 live births by 2030 is difficult to achieve.

Interventions to improve the quality of care around the intrapartum period are paramount for the achievement of a significant reduction in newborn mortality. These interventions encompass simple but lifesaving skills such as the resuscitation of newborns who fail to establish spontaneous breathing using a bag and mask [7,8]. However, evidence has shown that a significant number of healthcare providers (HCPs) do not have these necessary skills, and conventional training has been associated with skill decay over time as a result of little practice and inadequate refresher training [9,10]. For instance, evidence from the Helping Babies Breathe (HBB) program in Tanzania showed that after baseline training, HCPs acquired adequate skills which was also linked to an improvement in newborn morbidity and mortality [11]; however, skill evaluation six months later demonstrated a drop in skill performance whereby only 55.8% of participants passed evaluation compared to 87.1% immediately after the baseline training [12], which could be detrimental to newborn quality of care.

Low-dose high-frequency simulation-based training (LDHF-SBT)—a capacity-building approach that promotes maximal retention of clinical knowledge, skills, and attitudes through short, targeted in-service simulation-based learning activities, which are spaced over time and reinforced with structured, ongoing practice sessions on the job site—has been shown to improve the HCPs’ skills and confidence over time [13]. To leverage this, the Safer Births Bundle of Care (SBBC) was implemented in five regions with high newborn mortality in Tanzania [14]. The aim was to provide in-facility training of HCPs on newborn resuscitation using the second version of the HBB protocol [15]. Moreover, training was conducted by applying the low-dose high-frequency simulation method using improved simulation tools and using facility-generated clinical and training data for continuous quality improvement [14]. After baseline training, HCPs acquired neonatal resuscitation skills uniformly regardless of their previous training and workplaces [16]. Moreover, the endline evaluation of the SBBC intervention demonstrated a significant reduction in 24 h neonatal mortality by almost 40% [17]. However, for future sustainability and scalability, users’ acceptance of the intervention, internalization, and adoption of the intervention into their routine activities is paramount. It also depends on the user’s perception of the importance and practicability of the intervention [18,19]. However, the acceptability of this model of training and accompanying improved simulation tools is not well understood, especially in low-resource settings, including Tanzania.

Therefore, the main aim of this study was to assess the acceptability of low-dose, high-frequency, in situ simulation-based training for neonatal resuscitation and accompanying improved simulation tools among HCPs in two selected regions under SBBC intervention.

## 2. Materials and Methods

### 2.1. Study Sites and Population

This was a cross-sectional study conducted among HCPs working in labor wards and obstetric theaters in facilities under SBBC intervention in the Geita and Shinyanga regions. Health facilities for SBBC intervention were selected purposefully based on the number of births and rates of maternal and newborn morbidity and mortality. Additional inclusion criteria were the ability to provide comprehensive emergency obstetric and newborn care (CEmONC), which include the ability to perform basic neonatal resuscitation [20]. Twelve facilities were selected (six from each region). Among these, five were health centers (first referral level), five were district hospitals (second referral level), and two were regional referral hospitals (third referral level). In the Tanzania context, patients are managed or referred to the next referral level, as these have the capacity to provide emergency obstetric and newborn care.

Healthcare providers included nurses/midwives and doctors. In the Tanzania context, nurses/midwives with two years of formal training are awarded a certificate for their training; those with three years of training are awarded a diploma; and those with four or more years of formal training are awarded a bachelor’s degree. However, all are trained in basic competencies for midwifery practice. For this study, doctors were registered medical practitioners regardless of their level of training.

Inclusion criteria included the following: All HCPs meeting the following criteria were enrolled, including participated in the baseline training, did not shift from the labor ward/obstetric theater to other departments after the baseline training, and participated in LDHF-SBT for neonatal resuscitation.

### 2.2. Description of SBBC Interventions

The SBBC is a stepped-wedge cluster implementation study that aims to reduce maternal and newborn deaths around the time of delivery in five selected regions in Tanzania [14]. Interventions include skills and competency development among healthcare providers through in situ simulation-based training using improved training tools and clinical tools for effective monitoring of labor and guided neonatal resuscitation [14,21].

Tools for neonatal resuscitation training were co-developed in the Safer Births Consortium and produced by Laerdal Global Health (Stavanger, Norway) and aimed to save more lives at birth [21]. It included the following tools:

***NeoNatalie Live***: This is a newborn “smart” simulator which is connected to a tablet or phone by Bluetooth. It provides feedback to the learner regarding their skills performance in resuscitation, such as ventilation pressure, rate, effective mask seal, and positioning of the neck of the “baby.” This enables the trainer to identify areas for improvement.

***Upright bag and mask***: An upright bag and mask have an ergonometric design for effective mask seal and effective ventilation without the ventilator developing fatigue easily.

**Figure 1:**
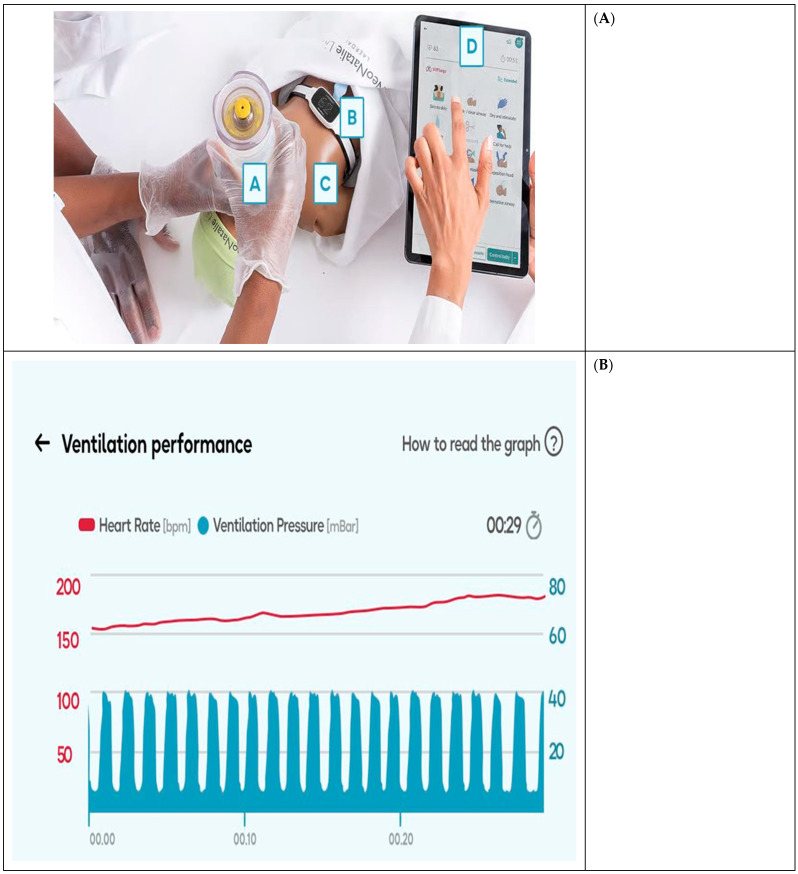
Improved tools for clinical and neonatal resuscitation training (photos by Laerdal Global Health). (**A**) Improved tools for clinical and neonatal resuscitation training: A = Upright bag and mask; B = NeoBeat; C = NeoNatalie Live; D = Tablet screen. (**B**) Excerpt from the tablet screen.

***NeoBeat***: This is a newborn heart rate meter that uses dry-electrode electrocardiography (ECG) technology to easily pick up and display the newborn’s heart rate. It is used instead of the stethoscope to guide the learner on the effectiveness of the ventilation process. Improved clinical and simulation tools are shown in Figure 1.

Additionally, the bundle utilized routine data generated in the health facilities to identify training and clinical care gaps as part of continuous quality improvement. The cascade of training under SBBC and mentorship activities was described in the SBBC protocol [14].

### 2.3. Data Collection Methods

Data collection was conducted one year after initial training and engagement in LDHF-SBT for neonatal resuscitation. Baseline training was conducted in facilities in the Geita and Shinyanga regions in December 2021 and in Shinyanga in February 2022, followed by in situ LDHF-SBT, and data collection was conducted in February and March 2023 in Geita and Shinyanga facilities, respectively. A total of 373 participants working in the labor ward were trained at baseline and enrolled in LDHF-SBT; however, only 227 participants met the criteria to participate in the acceptability assessment. The theoretical framework of an acceptability assessment of a healthcare intervention approach was used to assess acceptability [22,23]. This framework comprehensively assesses the acceptability of healthcare interventions in seven different constructs, which are based on cognitive and emotional responses which are likely to influence acceptability and engagement in the intervention.

The seven constructs in this theoretical framework are as follows: affective attitude of the participants toward the intervention; the burden of participating in the intervention; ethicality of the intervention; coherence; opportunity cost; perceived effectiveness; and self-efficacy [23]. Descriptions of the constructs are shown in Table 1.

A standardized five-point Likert scale questionnaire [24] based on the framework was self-administered to participants to self-evaluate their acceptance of LDHF-SBT for neonatal resuscitation. The assessment scale in each construct category ranged from 5 = Strongly agree; 4 = Agree; 3 = Neutral; 2 = Disagree; and 1 = Strongly disagree. Data collected on the hard copy of the questionnaire was reviewed by the study team for completeness and consistency before entry into the Open Data Kit (ODK) by trained data entry clerks and saved into the secure central database at Haydom Lutheran Hospital.

### 2.4. Data Analysis

#### 2.4.1. Acceptability Assessment

Data analysis was conducted by the first author and reviewed by experienced statisticians from Haydom Lutheran Hospital and Muhimbili University of Health and Allied Sciences. The Statistical Package for Social Sciences version 23 (IBM Ink, Chicago, IL, USA) was used for statistical analysis. Social demographic characteristics of the study participants were summarized into a frequency distribution table with respective percentages. The responses from the Likert questionnaire in each theoretical construct category were also summarized as frequencies and corresponding percentages. Further, in each construct category, those who responded in favor of the intervention (“strongly agree” and “agree”) were classified as having accepted the intervention in that specific construct, while those who responded not in favor of the intervention in specific construct categories (“neutral,” “disagree,” and “strongly disagree”) were classified as not having accepted the intervention in that specific construct.

The overall acceptability of the intervention was assessed using the same procedure for responses in the overall statement on to what extent the intervention is acceptable to the participant. For overall acceptability, we dichotomized the responses, whereby those who responded “acceptable” and “completely acceptable” were regarded as having accepted the intervention, while those whose responses were “no opinion,” “unacceptable,” and “completely unacceptable” were regarded as not having accepted the intervention. The analysis followed the guide for analyzing data on acceptability using the theoretical framework of an acceptability assessment of a healthcare intervention [23].

#### 2.4.2. Factors Associated with the Perceived Burden and Opportunity Cost of the Intervention

After a general assessment of acceptability in each acceptability construct category, constructs with mixed responses were further analyzed to assess factors associated with acceptability in those specific construct categories. Therefore, factors associated with the perceived burden of the intervention and opportunity cost were assessed using a modified Poisson regression model. The analysis was conducted in two steps: First, a bivariate analysis was performed with each independent variable separately. Crude prevalence ratios (CPR) and their respective 95% confidence intervals were computed. Second, independent variables with a *p*-value of less than or equal to 0.2 were included together in the multivariable model. Adjusted prevalence ratios (APR) and respective 95% confidence intervals were computed. Variables with a *p*-value of less than or equal to 0.05 were considered to be independently associated with the perceived burden and opportunity cost.

#### 2.4.3. The Association Between Perceived Burden, Opportunity Cost of the Intervention, and Frequency of Individual Practice

To assess whether the perceived burden and opportunity cost affected participation in LDHF-SBT, average individual practices per month were calculated for every participant. Those with averages of more than or equal to 5 individual practices per month were considered to have adequate practice. The association between average individual practices per month and the perceived burden and opportunity cost of the intervention were assessed using a chi-square test or Fisher’s exact test, depending on the expected frequency in each cell in the two-by-two table. A chi-square test result with a *p*-value of less than or equal to 0.05 was considered statistically significant. We present findings with the APR with 95% confidence intervals for each independent variable.

## 3. Results

All 227 participants who met the criteria agreed to participate and were enrolled in the study with a mean age of 35 (±8) years. Among these, 148 (65.2%) were females. Participants in the age group between 31 and 40 years formed the majority of the study participants (54.2%), and the majority cadre, 202 (89%), were nurses. Other demographic characteristics of study participants are summarized in Table 2.

### 3.1. Acceptability of LDHF-SBT Using Improved Tools

Overall, 223 (98.2%) reported that they accepted the intervention compared to 4 (1.8%) who were not in favor of the intervention. However, in the perceived burden construct, 202 (88.9%) reported that they made significant efforts to participate in the simulation training sessions. Additionally, with regard to the opportunity cost construct, 35 (15.4%) reported that engaging in LDHF-SBT interfered with other priorities. Other constructs that demonstrated positive acceptability among healthcare workers included the intervention being fair (96.9%), understanding the intervention (99.1%), the effectiveness of the intervention (99.1%), and the confidence of healthcare workers to participate in the intervention (99.6%). All acceptance levels in other construct categories are summarized in Table 3.

On assessing factors associated with the perceived burden of engaging in LDHF-SBT, no factor was significant at a *p*-value of less than or equal to 0.2 (Table 4). Therefore, further analysis to determine adjusted prevalence ratio was not performed.

On assessing factors associated with the opportunity cost of LDHF-SBT at the bivariate level, the region, sex of participants, and health facility level were associated with the opportunity cost of the intervention (the response that the intervention interfered with other important activities). However, on the multivariate model only the health facility level was significantly associated with the perceived cost of the intervention (Table 5).

### 3.2. Association Between the Perceived Burden of the Intervention and Average Individual Neonatal Resuscitation Practice per Month

On assessment of whether the perceived burden of engaging in LDHF-SBT affected the frequency of training, most of the participants, 192 (84.6%), had an average individual practice between 0 and 4 per month. However, the perceived burden of intervention did not affect the frequency of individual practice (Fisher’s test = 0.003; 0.63) as shown in Supplemental Table S1.

### 3.3. Association Between Perceived Opportunity Cost of the Intervention and Average Individual Neonatal Resuscitation Practice per Month

Similar to the perceived burden, there was no significant relationship between perceived opportunity cost and individual practice per month (Fisher’s test = 0.96; *p* = 0.47) as shown in Supplemental Table S2.

## 4. Discussion

In this study, we aimed to assess the acceptability of low-dose high-frequency in situ simulation-based training for neonatal resuscitation using improved simulation tools among healthcare providers in selected facilities under an SBBC intervention in two regions in Tanzania. Overall, the majority of participants (98.2%) accepted the intervention. However, on assessing acceptability in specific acceptability constructs, 91.2% reported that the intervention increased the burden of work-related activities, while 17.2% believed that the intervention interfered with other equally important activities. Moreover, working at a health center was independently associated with the perceived opportunity cost of the intervention. Nevertheless, the perceived burden and opportunity cost did not affect engagement in individual LDHF-SBT practices.

To the best of our understanding, this is the first study to assess the acceptability of LDHF-SBT for neonatal resuscitation using a structured theoretical framework for the assessment of health interventions. Even though most previous studies assessed the acceptability of this model using qualitative approaches, with limitations in the quantification of the acceptability levels, the overall findings were similar to our study, indicating a high level of acceptability [25,26,27,28]. Moreover, similar to our study findings, the acceptability of this model of neonatal resuscitation training was anchored on its ability to provide room for continuous improvement in knowledge, skills, team communication, and the quality of neonatal care [25,26,27,28]. In addition, although the model of training was similar, the tools used for training were slightly different. The tools used in our study were improved to increase the quality of practice through automated feedback from a newborn simulator (NeoNatalie Live), a newborn heart rate meter (NeoBeat), and an upright bag and mask with improved ergonomics for effective ventilation [14].

In this study, the perceived burden and opportunity cost of the intervention had mixed responses, whereby a significant number of participants perceived that the intervention increased their workload and interfered with other equally important activities. Nevertheless, this perception did not hinder them from participating in individual practice; they continued with practice despite feeling that it increased their workload. This could be linked to affection, coherence, and perceived effectiveness of the intervention, which is reflected by participants’ responses in these specific constructs. Moreover, earlier findings from our previous study on healthcare workers’ perception of SBBC support linkage of continuing training to the perceived effectiveness and relevancy of LDHF-SBT using improved tools [29].

We found that participants from the Geita region and those working in lower-level facilities were more likely to report that the intervention interfered with other equally important activities. Even though the amount of routine workload in the labor ward (deliveries) was not assessed in this study, it could be highly related to this feedback from participants. This is supported by our previous study on the implementation experience of the SBBC intervention, which showed that the Geita region had the highest number of deliveries per HCP compared to other regions [30]. Furthermore, overall, lower facility levels had higher deliveries compared to top-level facilities, which reflects a higher workload in these facilities [30]. Similar findings were also found in a recent study conducted in Uganda, which has a similar setting to Tanzania, where low staffing and workload were identified as significant barriers to LDHF-SBT [31]

The main strength of our study is anchored in the fact that there has been a great deal of in situ training in SBBC, and thus, we are in a unique position to study acceptability and burden associated with the training. Moreover, we used the validated theoretical framework for assessing health interventions, which comprehensively assesses the acceptability of the intervention in different constructs and provides room for quantification of the acceptability levels. This provides easy comparability with other similar studies using the same framework. Additionally, the study was conducted while participants were continuing with the intervention; this provides a timely assessment of perception and experiences and reduces recall bias. Nevertheless, our study was limited by the high mobility of HCPs who participated in the initial training, as some were relocated to other departments or to other health facilities, and therefore, they could not participate in this study. In addition, our study was a cross-sectional study, which is prone to antecedent-consequent bias. However, we minimized this bias by enrolling participants who had been involved in LDHF-SBT from baseline up to one year of implementation.

## 5. Conclusions

The acceptability of in-situ LDHF-SBT for neonatal resuscitation using improved training tools was high among healthcare workers. However, increased workload by engaging in regular practices and conflicting routine activities was perceived as a potential threat to engagement in regular simulation training. Nevertheless, this perceived threat did not stop participants from engaging in regular practices, which signifies healthcare workers’ confidence and perceived importance of the intervention in improving their skills and the quality of newborn care, with a subsequent reduction in newborn mortality.

For the successful scale-up and sustainability of LDHF-SBT for neonatal resuscitation, an equitable distribution of healthcare workers in labor wards in relation to workload is paramount. Furthermore, qualitative research among healthcare workers is needed for an in-depth understanding of barriers and facilitators of LDHF-SBT for neonatal resuscitation and to further guide effective large-scale implementation of the intervention.

## Figures and Tables

**Table 1:** Description of the theoretical framework for the acceptability assessment of the healthcare intervention.

Construct	Description
1. Affective attitude	Describes how a participant feels about the intervention.
2. Burden	The amount of effort participants need to put in to participate in the intervention, e.g., time, cognitive efforts, or expenses.
3. Ethicality	The extent to which the intervention is a good fit with participants’ values/ethical system and if the intervention is morally acceptable and meets the ethical standards of the participants’ environment.
4. Intervention coherence	The extent to which the participants understand the intervention and how it works.
5. Opportunity cost	The extent to which benefit, profits, or value must be given up to engage in the intervention.
6. Perceived effectiveness	The extent to which the intervention is perceived as likely to achieve its purpose.
7. Self-efficacy	The participant’s confidence that they can perform the activities/behavior required to participate in the intervention.

**Table 2:** Baseline demographic characteristics of study participants (N = 227).

Participants’ Characteristics	Frequency	Percent
**Region**		
Geita	127	55.9
Shinyanga	100	44.1
**Healthcare facility level**		
Health center	85	37.4
District hospital	71	31.3
Regional referral hospital	71	31.3
**Sex**		
Male	79	34.8
Female	148	65.2
**Age (years)**		
20–30	61	26.9
31–40	123	54.2
More than 40	43	18.9
**Level of education**		
Certificate	93	41.0
Diploma	118	52.0
Degree	16	7.0
**Cadre of the healthcare provider**		
Nurses	202	89
Doctors	25	11
**Working experience (years)**		
Less than 1	9	4.0
1–5	76	33.5
More than 5	142	62.6
**Experience working in the labor ward (years)**		
<1	19	8.4
1–5	117	51.5
>5	91	40.1
**History of previous experience in HBB training**		
Yes	218	96
No	9	4

**Table 3:** Acceptability in specific constructs (N = 227).

S/N	Construct Category	Response	Frequency (%)	Dichotomized Acceptability	
				**Yes**	**No**
**1**	**Affective attitude (how the participant feels about the intervention)**				
	Do you like LDHF-SBT using innovative tools?	Strongly dislike	2 (0.9)		
		No opinion	2 (0.9)	223 (98.2)	4 (1.8)
		Like	36 (15.9)		
		Strongly like	187 (82.4)		
**2**	**Burden (perceived amount of effort required to participate in the intervention)**				
	How much effort did it take to engage in LDHF-SBT using innovative tools?	No effort	3 (1.3)		
		Little effort	17 (7.5)		
		No opinion	5 (2.2)	20(8.8)	207 (91.2)
		A lot of effort	127 (55.9)		
		Huge effort	75 (33.0)		
**3**	**Ethicality (the extent to which the intervention is a good fit (fairness) with the participant’s value system)**				
	How fair is the LDHF-SBT using innovative tools for you who participated in the training and the patient’s care?	No opinion	7 (3.1)		
		Fair	201 (88.5)	220 (96.9)	7 (3.1)
		Very fair	19 (8.4)		
**4**	**Intervention coherence (the extent to which the participant understands the intervention and how it works)**				
	It is clear to me how the LDHF-SBT using innovative tools will help to improve my skills in neonatal resuscitation.	No opinion	2 (0.9)		
		Agree	102 (44.9)		
		Strongly agree	123 (54.2)	225 (99.1)	2 (0.9)
5	**Perceived effectiveness (the extent to which the intervention has achieved its purpose)**				
	The LDHF-SBT, using innovative tools, has improved my skills in neonatal resuscitation and improved the quality of care.	No opinion	2 (0.9)		
		Agree	55 (23.8)	225 (99.1)	2 (0.9)
		Strongly agree	170 (74.4)		
6	**Self-efficacy (the participants’ confidence that they can participate in the intervention)**				
	How confident do you feel about engaging in LDHF-SBT using innovative tools?	No opinion	1 (0.4)		
		Confident	129 (56.8)	226 (99.6)	1 (0.4)
		Very confident	97 (42.7)		
**7**	**Opportunity cost (benefits, profits, or value that were given up by engaging in the intervention)**				
	Engaging in the LDHF-SBT using innovative tools interfered with my other important priorities.	Strongly disagree	101 (44.5)		
		Disagree	87 (38.3)		
		No opinion	4 (1.8)	188 (82.9)	39 (17.2)
		Agree	23 (10.1)		
		Strongly agree	12 (5.3)		
8	**General acceptability**				
	How acceptable was the LDHF-SBT using innovative tools to you?	Completely unacceptable	2 (0.9)		
		No opinion	2 (0.9)	223 (98.2)	4 (1.8)
		Acceptable	65 (65)		
		Completely acceptable	158 (69.6)		

**Table 4:** Factors associated with perceived burden of involvement in LDHF-SBT for neonatal resuscitation (N = 227).

Variable		CPR	95% CI	*p*-Value
**Region**				
	Geita	1	0.8–1.2	0.9
	Shinyanga	Ref.		
**Facility level**				
	Health center	1	0.8–1.3	0.9
	District hospital	1	0.8–1.3	0.9
	Regional referral hospital	Ref.		
**Sex**				
	Male	0.9	0.8–1.2	0.91
	Female	Ref.		
**Level of Education**				
	Certificate	1.1	0.7–1.6	0.74
	Diploma	1.1	0.7–1.5	0.8
	Degree	Ref.		
**Age group (years)**				
	20–30	1	0.8–1.3	0.99
	31–40	0.9	0.8–1.3	0.88
	More than 40	Ref.		
**Cadre**				
	Nurses/midwives	1.1	0.8–1.4	0.78
	Clinicians (doctors)	Ref.		
**Work experience (years)**				
	Less than a year	0.9	0.7–1.5	0.78
	1–5 years	1	0.8–1.2	0.89
	More than 5 years	Ref.		
**Experience working in the labor ward (years)**				
	Less than a year	1.0	0.7–1.4	0.98
	1–5 years	1.0	0.8–1.2	0.83
	More than 5 years	Ref.		

**Table 5:** Factors associated with the perceived opportunity cost of engaging in LDHF-SBT for neonatal resuscitation (N = 227).

Variable		PR	95% CI	*p* Value	APR	95% CI	*p*-Value
**Region**							
	Geita	2	1.1–3.8	0.04	1.9	1–3.6	0.06
	Shinyanga						
**Facility level**							
	Health center	2.2	0.9–5	0.08	1.9	0.8–4.7	0.15
	District hospital	2.8	1.2–6.8	0.02	2.9	1.2–6.8	0.02
	Regional referral hospital	Ref.					
**Sex**							
	Male	1.8	1.1–3.1	0.05	1.5	0.8–2.6	0.2
	Female	Ref.					
**Level of Education**							
	Certificate	0.6	0.2–2	0.44	1.1	0.5–2.1	0.97
	Diploma	1.1	0.4–3.3	0.82	1.1	0.6–2.1	0.84
	Degree	Ref.					
**Age group (years)**							
	20–10	0.8	0.3–1.9	0.6	0.8	0.5–1.4	0.58
	31–40	0.9	0.5–2	0.92	0.9	0.6–1.3	0.64
	More than 40	Ref.					
**Cadre**							
	Nurses/midwives	0.6	0.3–1.1	0.11	0.7	0.4–1.5	0.42
	Clinicians (doctors)	Ref.					
**Work experience (years)**							
	Less than a year	1.3	0.4–4.5	0.72			
	1–5 years	0.9	0.5–1.7	0.72			
	More than 5 years	Ref.					
**Experience working in the labor ward (years)**							
	Less than a year	1.1	0.3–3.5	0.87			
	1–5 years	1.4	0.7–2.6	0.32			
	More than 5 years	Ref.					

## Data Availability

Data can be available with prior written request to the Institutional Review Board of the Muhimbili University of Health and Allied Sciences.

## Supplementary Materials

The following supporting information can be downloaded at https://www.mdpi.com/article/10.3390/children12091150/s1, Table S1: relationship between perceived burden of the intervention and individual practice of neonatal resuscitation per month; Table S2: relationship between perceived burden of engaging in LDHF-SBT and individual practice per month.

## Abbreviations

The following abbreviations are used in this manuscript:

CEmONC	Comprehensive Emergency Obstetric and Newborn Care
HBB	Helping Babies Breathe
HCP	Healthcare Provider
LDHF-SBT	Low-Dose High-Frequency Simulation-Based Training
ODK	Open Data Kit
SBBC	Safer Births Bundle of Care
SDG	Sustainable Development Goals
