# Supplementary material for: Acceptability Among Healthcare Providers of In Situ, Low-Dose, High-Frequency Neonatal Resuscitation Simulation Training Using Innovative Tools: Evidence from the Safer Births Bundle of Care
Source: Children (Basel). 2025 Aug 29;12(9):1150. doi: 10.3390/children12091150 (PMC12468043; doi:10.3390/children12091150)
Supplement: Supplementary file 1 [file children-12-01150-s001.zip › children-3764191-supplementary.pdf]

Supplemental Table S1: Relationship between perceived burden of the intervention and individual practice of neonatal resuscitation per month (N=227).

Reported efforts needed to engage in frequent training	Individual trainings per month		Total
	0-4 training	≥ 5 training	
No significant efforts	17 (85)	3(15)	20(100)
Significant efforts	175(84.5)	32(15.5)	207(100)
Total	192(84.6)	35(15.4)	227 (100)

Fisher's exact test=0.003; p=0.63

Supplemental Table S2: Relationship between perceived burden of engaging in LDHF-SBT and individual practice per month (N=227).

Engaging in LDHF-SBT for neonatal resuscitation interfered with other important activities	Individual training per month		Total
	0-4 training	≥ 5 training	
No	157(85.5)	31 (16.5)	118(100)
Yes	35(89.7)	4(13.5)	29(100)
Total	192(84.6)	35(15.4)	227 (100)

Fisher's exact test=0.96; p=0.47
